# Supplementary material for: Unveiling pelagic-benthic coupling associated with the biological carbon pump in the Fram Strait (Arctic Ocean)
Source: Nat Commun. 2025 Jan 20;16:840. doi: 10.1038/s41467-024-55221-x (PMC11747630; doi:10.1038/s41467-024-55221-x)
Supplement: Supplementary file 1 — Supplementary information [file 41467_2024_55221_MOESM1_ESM.pdf]

Supplementary Information

Unveiling pelagic-benthic coupling associated with the biological carbon pump in the Fram Strait (Arctic Ocean)

Simon Ramondenc[1,2*], Damien Eveillard[3,4], Katja Metfies[1], Morten H. Iversen[1,2], Eva-Maria Nöthig[1], Dieter Piepenburg[1], Christiane Hasemann[1], Thomas Soltwedel[1]

[1] Alfred Wegener Institute, Helmholtz Centre for Polar and Marine Research, Am Handelshafen 12, 27570 Bremerhaven, Germany

[2] MARUM, Center for Marine Environmental Sciences, University of Bremen, Leobener Strasse 8, 28359 Bremen, Germany

[3] Nantes Université, Ecole Centrale Nantes, CNRS, LS2N, F-44322 Nantes, France

[4] Research Federation for the Study of Global Ocean Systems Ecology and Evolution, FR2022/Tara Oceans GOSEE, F-75016, Paris, France

*** Correspondence:**
Simon Ramondenc (simon.ramondenc@awi.de)

Supplementary Text

1| Sample treatment

Pelagic sample. Before each deployment, the sampling cups on the sediment traps were filled with filtered seawater adjusted to a salinity of 40 PSU with NaCl and poisoned with mercury chloride ($HgCl_2$: final solution of 0.14%) to preserve the collected material in the sampling cups during deployment and after recovery. Once recovered, sediment trap samples were refrigerated at 4°C and stored in the dark until laboratory analyses. Before biogeochemical analysis of the material collected by the sediment traps, swimmers (*i.e.*, zooplankton actively swam into the trap) and sinkers (*i.e.*, zooplankton passively sink into the trap) larger than 0.5 mm were picked individually under microscope using soft forceps and gently rinsed with filtered seawater in order to be identified, counted, and grouped into six main groups (*i.e.*, Copepoda, Pteropoda, Amphipoda, Ostracoda, Foraminifera, Chaetognatha). The sinkers are included in the biogeochemical fluxes, as they represent naturally sinking particulate matter, unlike swimmers, which do not reflect natural sedimentation processes. Hereafter, the samples were divided by a wet splitting procedure as ⅛ volumetric splits. The subsamples were used to estimate biogeochemical measurements such as total particulate matter flux (TMP), particulate organic carbon (POC), particulate organic nitrogen (PON), biogenic silica (PbSi), calcium carbonate ($CaCO_3$; including pteropods), and carbon and nitrogen isotopes ($\partial^{13}C$ and $\partial^{15}N$). Methods and protocols used to measure all the listed biogeochemical fluxes are already provided by Lalande et al.[1,2] and Bauerfeind et al.[3]. In addition, environmental variables and climatic indices associated with each sample were obtained from the mooring system and satellite observation following the method described in Ramondenc et al.[4].

Benthic sample. Three samples from different cores of the same multicorer deployment were taken using plastic syringes with anterior cut-off ends (ø 1.2 and 2 cm) to estimate abiotic and biotic factors such as: water contents (H_2O), organic carbon contents (Corg), chlorophyll a and the bulk of chloroplastic pigments (chloroplastic pigment equivalents, CPE), particulate protein and phospholipid concentrations (Lipids), bacterial activities, bacterial numbers, the mean bacteria biomass per cell (MBC), and bacterial biomasses. A detailed description of methods for analyzing the water content and biogenic sediment compounds is provided by Soltwedel et al.[5]. Sediment cores were sectioned in 1 cm layers to estimate meiofauna abundance in the uppermost 5 cm of the sediments and fixed immediately with 4% buffered formaldehyde. Back in the laboratory, the meiofauna was washed, counted, and grouped into 11 main groups (*i.e.*, Nematoda, Copepoda, nauplii, Ostracoda, Polychaeta, Rotifera, Gastrotricha, Kinorhyncha, Bivalvia, Tardigrada and "Others" summarising all other taxa) according to Soltwedel et al.[6].

Illumina-Sequencing 18S rDNA & Sequence analyses. An Illumina MiSeq sequencer (Illumina, San Diego, CA, USA) with MiSeq v3 reagent kit was used for sequencing, which produced 2 x 300 paired-end amplicon reads. For demultiplexing and fastq sequencing raw file generation the Miseq build-in "Generate FASTQ" workflow was utilized. The sequences of the PCR primer pair were detected at 20% mismatch error rate and removed with *cutadapt* (v. 2.8)[7]. Sequence pairs were discarded, if at least one primer sequence could not be detected at the 5'-ends of the amplicon sequences. For further sequence processing the *DADA2* R package (v. 1.18.0)[8] was used. As read quality usually drops towards the 3'-end, the reads were trimmed after 250-265bp (forward reads) and 230-240bp (reverse reads), respectively. The trimming lengths were set after visual inspection of the read quality plots. Additionally, utilising the Phred quality scores provided alongside the called bases, sequence pairs were removed from the data set, if the expected base prediction error per sequence exceeded certain values which were set proportionally to the respective lengths after trimming. Incorporating the Phred quality scores, error rates were learned for the forward and the reverse read files separately and for each sample file pool originating from the same sequencing run separately as well. Utilizing the calculated error rates, the sequences were denoised by the "Divisive Amplicon Denoising Algorithm" (DADA2). Following, paired-end reads were merged with a minimum overlap of 25 bp disallowing

any mismatches in the overlapping region. An ASV table combining denoised amplicon sequence variants of each sample pool was created, and chimeras were predicted by VSEARCH in de novo mode with default settings and removed from the sample files. Taxonomic assignment of the ASVs was performed using the reference databases PR2 (v4.12.0)[9] with default settings.

3| Individual lineage analyses

Independently to the global structure of the pelagic and benthic communities, a sparse partial least squares (sPLS)[10] in regression mode was performed *via* the R package *mixOmics*[11] on benthic and pelagic CLR-transformed ASVs abundance matrices separately. This method was useful to relate and predict community composition and its environmental traits without a potential clustering effect. The number of ASV sequences and environmental variables to be kept in the model for each component was set according to the *tune.spls* function implemented in the *mixOmics* packages. Following the percentage of variables explained in the upstream principal component analysis, the number of components has been set to 3 and 4 in the sPLS analyses for the pelagic and benthic datasets, respectively. sPLS confirmed the findings from the WGCNA approach, identifying several plankton lineages whose relative ASV abundance correlated with carbon flux in the water column and carbon content in the sediment core. These lineages included diatoms (i.e., Bacillariophyta), dinoflagellates (i.e., Peridiniales, Gymnodiniales), Haptophytes (i.e., Phaeocystales), radiolarians (i.e., Chaunacanthida), and metazoans like Aphragmophora.

4| Method limitations & uncertainties

During the past decades, the so-called "-omics" revolution made major advances in our understanding of the role of marine diversity in biogeochemical cycles. Our study provides the first analysis of pelagic-benthic coupling based on a unique long-term observation of eDNA from the subpolar region. Nevertheless, a significant part of the sequences recorded in the samples remains unknown (14% and 7% of benthic and pelagic ASVs, respectively, were not classified beyond the kingdom level), even though the available taxonomic assignment databases for DNA sequences have been improved and updated regularly in recent years. Inherent to meta-omics research, filling the gap and reducing this uncertainty requires time and collective scientific effort. Another major issue in the metabarcoding approach is the genetic markers (*e.g.*, V4 region) used to sequence DNA, which can potentially influence Metazoan identification, eukaryotic community compositions and overall detection success. The V4 region of the 18S rDNA gene was specifically selected as the most suitable marker for this long-term study due to its reliability in capturing microbial diversity over time and its ability to provide a comprehensive representation of eukaryotic microbial biodiversity. Recent publications have highlighted the advantages of using the V4 region for biodiversity assessments, especially when compared to or combined with optical surveys for both phytoplankton and zooplankton[12, 13]. This evidence supported the decision to focus on the V4 marker, to achieve an accurate and detailed representation of microbial communities over the extended time series. Moreover, the high correlation between Chaetognatha counted under the microscope and the pelagic subnetwork S3, mainly composed of Aphragmophora, provided confidence that the DNA sequences were consistent and matched the organisms present in the samples. Only a few zooplanktonic groups commonly recorded in the sediment traps as swimmers were undetected in the 18S assemblage (*e.g.*, Amphipoda and Ostracoda). Swimmers were picked out of the trap samples but chaetognaths were commonly found in multiple pieces which potentially provided the highest genetic material amount. However, the choice of genetic markers can introduce significant biases in the biodiversity detected through eDNA metabarcoding[14, 15]. As sequencing costs decline and refined primer-sets become available, a multi-primer approach has become feasible. This strategy enhances the accuracy of the taxonomic annotation of raw sequences and helps minimize the effects of marker-specific limitations on biodiversity estimates[14, 15]. Another unknown and potentially altering 18S assemblage is the eDNA degradation during the sampling period and, especially, the efficiency of mercury chloride in preserving soft bodies or small cells. According to a recent publication, the mercury chloride used in sediment traps to preserve organic matter does not severely change the

nucleotide composition of 18S RNA[16, 17]. Furthermore, previous studies have demonstrated that replicating PCRs and sequencing yield highly consistent results[16], we acknowledge that including replicates is important for strengthening our findings, particularly for rare taxa. In this study, replicate sequencing was not included; however, we encourage its use in future research to enhance the robustness of results. The extended timeframe of our research further ensures a clear distinction between technical and natural variability, supporting the validity of our approach.

The phenology of marine species (*e.g.*, seasonal changes), the settling particles (*e.g.*, aggregation with phytodetritus), the sea-ice condition (*e.g.*, stratification, productivity), and the physical dynamics (*e.g.,* lateral advection, mesoscale structures) are all factors that may induce a large dissimilarity of ASVs composition between samples in the region. Hence, two different sampling strategies were adopted following the ecosystem and its inherent variability. While the high spatial resolution uncertainties in the upper water column are supposed to shape planktonic communities at (sub-)mesoscale, we decided to allocate more effort to the temporal resolution assuming that the inter and intra-annual changes were the most relevant factors determining pelagic community interactions. The deep Arctic Ocean, especially benthic ecosystems, is assumed to be stable with little seasonal variation[18] but highly dependent on the spatial variability for shaping benthic community interactions. For this reason, a high spatial resolution was chosen rather than a high temporal resolution.

From a statistical perspective, we proposed the graph-alignment method, especially used to compare protein interaction data, as a powerful tool to represent and describe pelagic-benthic coupling. Nevertheless, several statistical methods used in the present study are based on correlation association, which does not necessarily imply causality.

Supplementary Figures

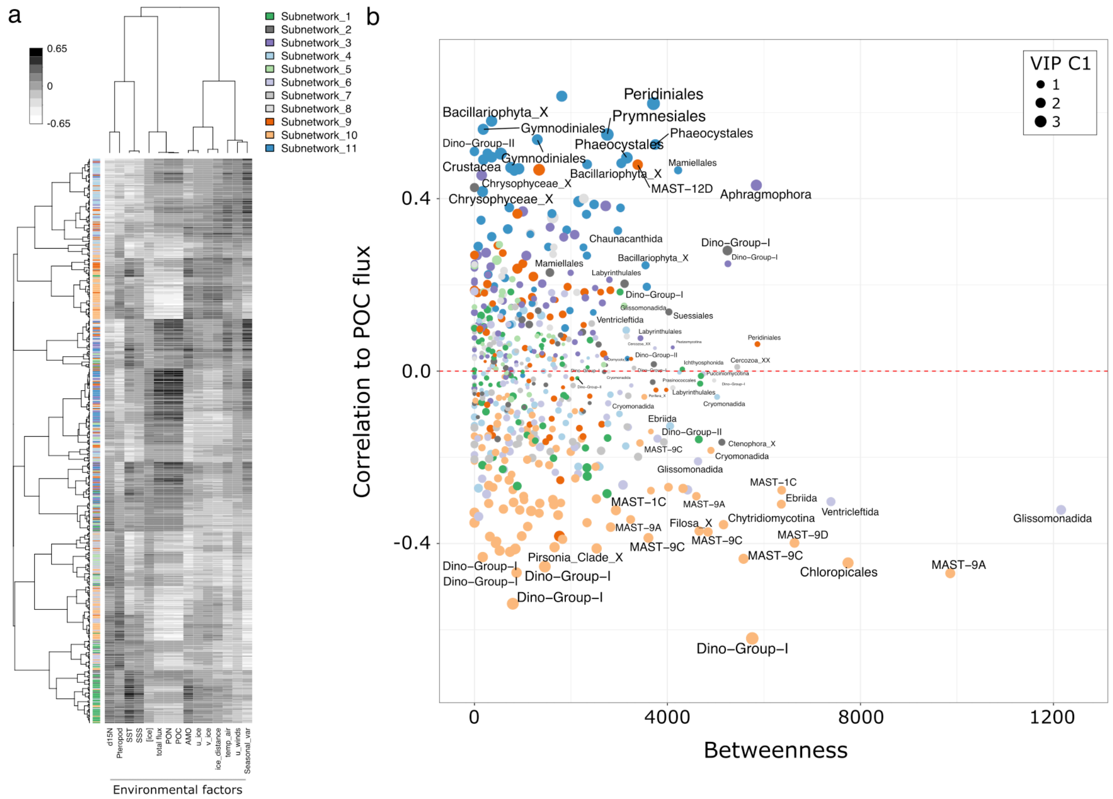

Figure S1. Key pelagic lineages associated with the POC flux. (a) Sparse Partial Least Squares analysis showing the correlations between lineages and environmental data. Colors along the clustered heatmap represent the subnetworks previously identified by WGCNA. (b) Global view of the central lineages in the planktonic network (*i.e.*, betweenness), correlated and predict (*i.e.*, VIP score) the carbon export.

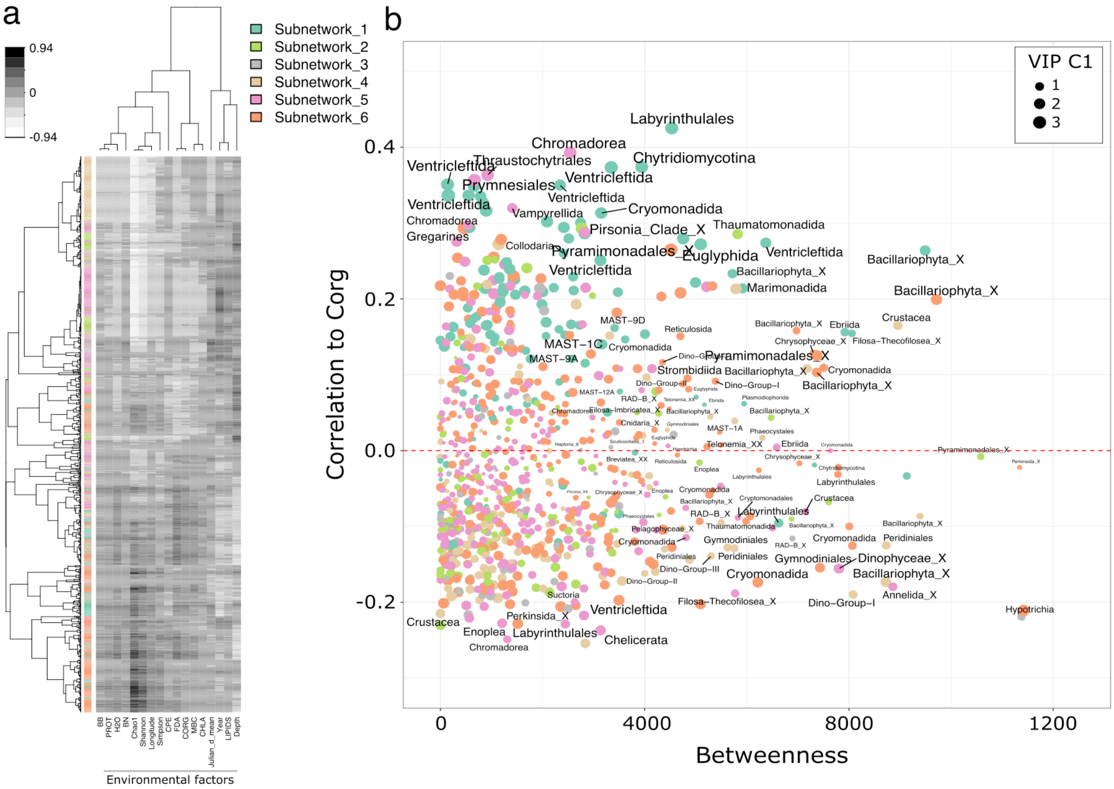

Figure S2. Key benthic lineages associated with the carbon organic content. (a) Sparse Partial Least Squares analysis showing the correlations between lineages and environmental data. Colors along the clustered heatmap represent the subnetworks previously identified by WGCNA. (b) Global view of the central lineages in the benthic network (*i.e.*, betweenness), correlated and predict (*i.e.*, VIP score) the carbon export.

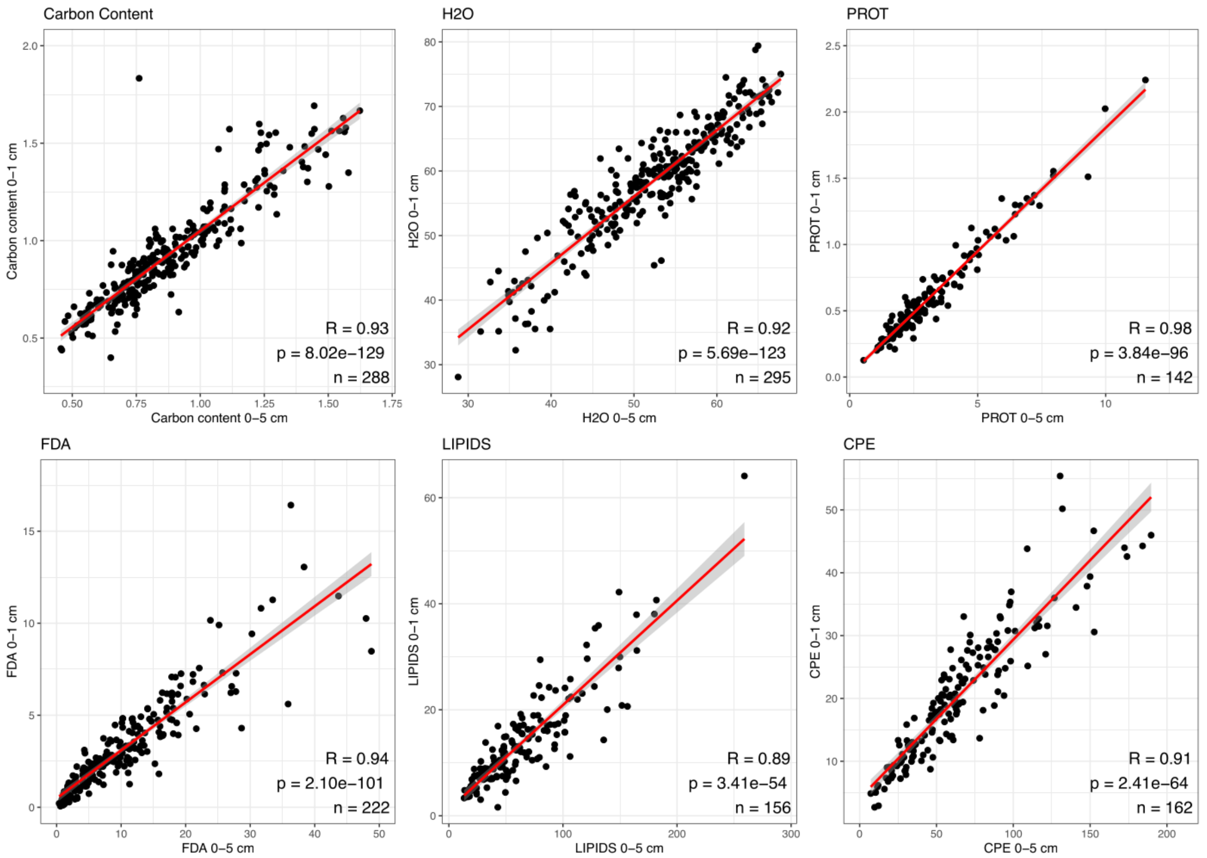

Figure S3. Linear regression analysis of core parameters (carbon content, water content H_2O, particulate proteins PROT, bacterial activities FDA, phospholipid concentration Lipids and Chlorophyll pigment equivalents CPE) comparing data from the top 5 cm versus the first 1 cm of sediment. The p-values represent the results of Pearson correlation tests, indicating the statistical significance of the correlations. R values denote the correlation coefficients, reflecting the strength and direction of the relationships between the two depths for each core parameter, while the shaded area represents the 95% confidence interval.

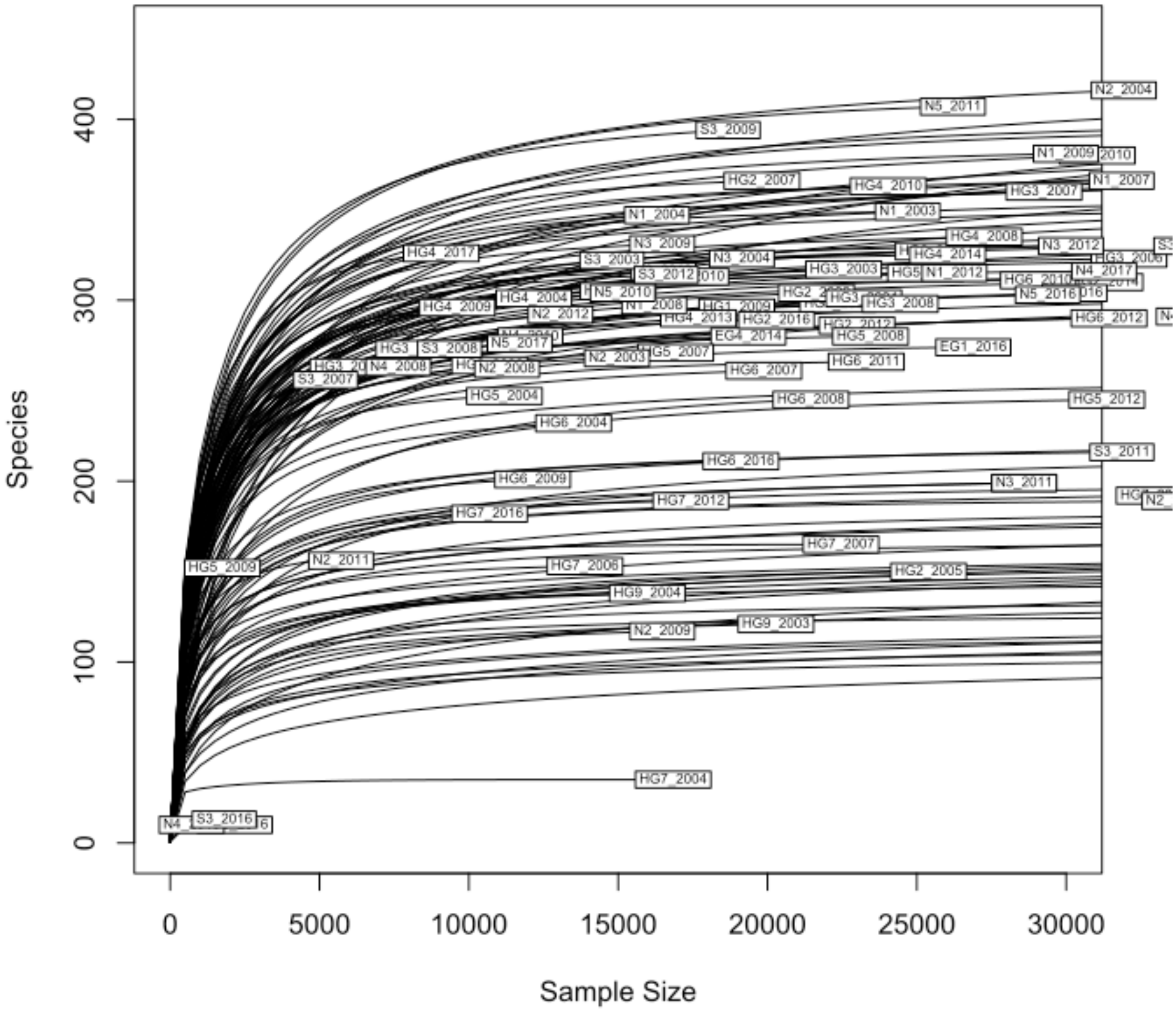

Figure S4. Rarefaction curves of sediment core samples. The curves illustrate the relationship between the sample size and the observed diversity of sediment core samples.

Supplementary References

1. Lalande C, Bauerfeind E, Nöthig E-M, Beszczynska-Möller A. Impact of a warm anomaly on export fluxes of biogenic matter in the eastern Fram Strait. *Prog Oceanogr* **109**, 70-77 (2013).
2. Lalande C, Nöthig E-M, Bauerfeind E, Hardge K, Beszczynska-Möller A, Fahl K. Lateral supply and downward export of particulate matter from upper waters to the seafloor in the deep eastern Fram Strait. *Deep Sea Research Part I: Oceanographic Research Papers* **114**, 78-89 (2016).
3. Bauerfeind E*, et al.* Particle sedimentation patterns in the eastern Fram Strait during 2000–2005: Results from the Arctic long-term observatory HAUSGARTEN. *Deep Sea Research Part I: Oceanographic Research Papers* **56**, 1471-1487 (2009).
4. Ramondenc S*, et al.* Effects of Atlantification and changing sea-ice dynamics on zooplankton community structure and carbon flux between 2000 and 2016 in the eastern Fram Strait. *Limnol Oceanogr*, (2022).
5. Soltwedel T*, et al.* Natural variability or anthropogenically-induced variation? Insights from 15 years of multidisciplinary observations at the arctic marine LTER site HAUSGARTEN. *Ecological Indicators* **65**, 89-102 (2016).
6. Soltwedel T, Grzelak K, Hasemann C. Spatial and temporal variation in deep-sea meiofauna at the LTER Observatory HAUSGARTEN in the Fram Strait (Arctic Ocean). *Diversity* **12**, 279 (2020).
7. Martin M. Cutadapt removes adapter sequences from high-throughput sequencing reads. *EMBnet journal* **17**, 10-12 (2011).
8. Callahan BJ, McMurdie PJ, Rosen MJ, Han AW, Johnson AJA, Holmes SP. DADA2: High-resolution sample inference from Illumina amplicon data. *Nature methods* **13**, 581-583 (2016).
9. Guillou L*, et al.* The Protist Ribosomal Reference database (PR2): a catalog of unicellular eukaryote small sub-unit rRNA sequences with curated taxonomy. *Nucleic acids research* **41**, D597-D604 (2012).
10. Shen H, Huang JZ. Sparse principal component analysis via regularized low rank matrix approximation. *Journal of multivariate analysis* **99**, 1015-1034 (2008).
11. Rohart F, Gautier B, Singh A, Lê Cao K-A. mixOmics: An R package for 'omics feature selection and multiple data integration. *PLoS computational biology* **13**, e1005752 (2017).
12. Weiß JF*, et al.* Unprecedented insights into extents of biological responses to physical forcing in an Arctic sub-mesoscale filament by combining high-resolution measurement approaches. *Sci Rep-Uk* **14**, 8192 (2024).
13. Weydmann-Zwolicka A, Dąbrowska AM, Mioduchowska M, Zwolicki A. Comparison of DNA metabarcoding and microscopy in analysing planktonic protists from the European Arctic. *Marine Biodiversity* **54**, 1-10 (2024).
14. Clarke LJ, Beard JM, Swadling KM, Deagle BE. Effect of marker choice and thermal cycling protocol on zooplankton DNA metabarcoding studies. *Ecol Evol* **7**, 873-883 (2017).
15. Zimmermann HH, Harðardóttir S, Ribeiro S. Assessing the performance of short 18S rDNA markers for environmental DNA metabarcoding of marine protists. *Environmental DNA* **6**, e580 (2024).
16. Metfies K*, et al.* Protist communities in moored long-term sediment traps (Fram Strait, Arctic)–preservation with mercury chloride allows for PCR-based molecular genetic analyses. *Frontiers in Marine Science* **4**, 301 (2017).
17. Wietz M*, et al.* The polar night shift: seasonal dynamics and drivers of Arctic Ocean microbiomes revealed by autonomous sampling. *ISME Communications* **1**, 76 (2021).
18. Sanders HL. Marine benthic diversity: a comparative study. *The American Naturalist* **102**, 243-282 (1968).
