# Supplementary material for: Unveiling pelagic-benthic coupling associated with the biological carbon pump in the Fram Strait (Arctic Ocean)
Source: Nat Commun. 2025 Jan 20;16:840. doi: 10.1038/s41467-024-55221-x (PMC11747630; doi:10.1038/s41467-024-55221-x)
Supplement: Supplementary file 2 — Reporting Summary [file 41467_2024_55221_MOESM2_ESM.pdf]

nature portfolio

Corresponding author(s): Ramondenc Simon

Last updated by author(s): Nov 27, 2024

Reporting Summary

Nature Portfolio wishes to improve the reproducibility of the work that we publish. This form provides structure for consistency and transparency in reporting. For further information on Nature Portfolio policies, see our Editorial Policies and the Editorial Policy Checklist.

Statistics

For all statistical analyses, confirm that the following items are present in the figure legend, table legend, main text, or Methods section.

n/a	Confirmed	
☐	☒	The exact sample size (*n*) for each experimental group/condition, given as a discrete number and unit of measurement
☐	☒	A statement on whether measurements were taken from distinct samples or whether the same sample was measured repeatedly
☐	☒	The statistical test(s) used AND whether they are one- or two-sided<br>*Only common tests should be described solely by name; describe more complex techniques in the Methods section.*
☒	☐	A description of all covariates tested
☒	☐	A description of any assumptions or corrections, such as tests of normality and adjustment for multiple comparisons
☐	☒	A full description of the statistical parameters including central tendency (e.g. means) or other basic estimates (e.g. regression coefficient) AND variation (e.g. standard deviation) or associated estimates of uncertainty (e.g. confidence intervals)
☒	☐	For null hypothesis testing, the test statistic (e.g. *F*, *t*, *r*) with confidence intervals, effect sizes, degrees of freedom and *P* value noted<br>*Give P values as exact values whenever suitable.*
☒	☐	For Bayesian analysis, information on the choice of priors and Markov chain Monte Carlo settings
☒	☐	For hierarchical and complex designs, identification of the appropriate level for tests and full reporting of outcomes
☒	☐	Estimates of effect sizes (e.g. Cohen's *d*, Pearson's *r*), indicating how they were calculated

Our web collection on statistics for biologists contains articles on many of the points above.

Software and code

Policy information about availability of computer code

Data collection	The raw sequences have been deposited in the European Nucleotide Archive (ENA) under the accession numbers PRJEB76183 for sediment cores and PRJEB74771 for sediment traps. All other data are publicly available, with access instructions provided in the main manuscript and the following GitHub repository: https://github.com/sramondenc/NCOMMS-24-22914_Pelagic_Benthic_Coupling_Arctic/
Data analysis	The code used to perform the pelagic and benthic network analyses are accessible at: https://github.com/sramondenc/NCOMMS-24-22914_Pelagic_Benthic_Coupling_Arctic/

For manuscripts utilizing custom algorithms or software that are central to the research but not yet described in published literature, software must be made available to editors and reviewers. We strongly encourage code deposition in a community repository (e.g. GitHub). See the Nature Portfolio guidelines for submitting code & software for further information.

Data

Policy information about availability of data

All manuscripts must include a data availability statement. This statement should provide the following information, where applicable:

- Accession codes, unique identifiers, or web links for publicly available datasets
- A description of any restrictions on data availability
- For clinical datasets or third party data, please ensure that the statement adheres to our policy

The raw sequences data generated in this study have been deposited in the European Nucleotide Archive (ENA) database under accession code PRJEB76183

[https://www.ebi.ac.uk/ena/browser/view/PRJEB76183] for sediment cores and PRJEB74771 [https://www.ebi.ac.uk/ena/browser/view/PRJEB74771] for sediment traps.

Research involving human participants, their data, or biological material

Policy information about studies with human participants or human data. See also policy information about sex, gender (identity/presentation), and sexual orientation and race, ethnicity and racism.

Reporting on sex and gender	n/a
Reporting on race, ethnicity, or other socially relevant groupings	n/a
Population characteristics	n/a
Recruitment	n/a
Ethics oversight	n/a

Note that full information on the approval of the study protocol must also be provided in the manuscript.

Field-specific reporting

Please select the one below that is the best fit for your research. If you are not sure, read the appropriate sections before making your selection.

☐ Life sciences ☐ Behavioural & social sciences ☒ Ecological, evolutionary & environmental sciences

For a reference copy of the document with all sections, see nature.com/documents/nr-reporting-summary-flat.pdf

Ecological, evolutionary & environmental sciences study design

All studies must disclose on these points even when the disclosure is negative.

Study description	This study explores how different plankton communities contribute to the transport of organic matter from the ocean surface to the deep sea. By analyzing 15 years of environmental DNA (eDNA) from the Fram Strait, we identified key organisms involved in carbon export, including the diatom Chaetoceros socialis, sea-ice diatoms, Radiolaria, and Chaetognatha. We found that C. socialis played a critical role in the amount of organic carbon reaching the seafloor. The study also highlights the surprising role of parasites in carbon flux and their potential influence on sedimentation and ecosystem functioning.
Research sample	Pelagic samples (n= 83) were collected using Kiel-type sediment traps moored at depths between 179 m and 280 m at the central HAUSGARTEN station from 2000 to 2012. Benthic samples (n=145) were gathered from the top five centimeters of seafloor sediments using a TV-guided multiple corer during annual summer cruises from 2003 to 2018. Unlike the pelagic sampling, benthic samples were taken from 19 HAUSGARTEN stations along longitudinal and latitudinal transects in the Fram Strait.
Sampling strategy	Pelagic samples were collected with Kiel-type sediment traps (KUM trap type K/MT 234) moored in the water column between 179 m and 280 m water depths at the central HAUSGARTEN station (HG-IV) from 2000 to 2012. Moorings and sediment traps were exchanged annually. For benthic samples, the top five centimeters of the seafloor sediments were sampled using a TV-guided multiple corer (TV-MUC; corer ø 10 cm; length 50 cm) during the annual summer cruises to the HAUSGARTEN observatory between 2003 and 2018. In contrast to the pelagic sampling, nineteen HAUSGARTEN stations were investigated along longitudinal (i.e., HG-I to HG-IX) and latitudinal transects (i.e., S3 to N5), passing by stations regularly covered by sea-ice (i.e., EG-I to EG-IV) in the western Fram Strait.
Data collection	Data collection for this study spanned 15 years and involved analyzing environmental DNA (eDNA) sequences (18S-V4) from settling and sedimented organic matter in the Fram Strait. All eDNA samples were collected by the Alfred Wegener Institute, under the expertise of Katja Metfies.
Timing and spatial scale	Pelagic samples were collected at depths between 179 m and 280 m at the central HAUSGARTEN station from 2000 to 2012. Benthic samples were collected during annual summer cruises from 2003 to 2018 at 19 HAUSGARTEN stations along longitudinal and latitudinal transects in the Fram Strait.
Data exclusions	No data exclusion
Reproducibility	As mentioned in the manuscript, we typically do not perform replicates for the entire dataset due to the extensive effort required for large time-series studies. However, findings from previous studies confirmed that replicates produce highly consistent results, even with minimal DNA quantities. This consistency supports the robustness of our results. Additionally, the long-term scope of our analysis allows us to distinguish genuine ecological patterns from any potential analytical variability.
Randomization	No experiement with groups

Blinding	No experiment with groups

Did the study involve field work? ☒ Yes ☐ No

Field work, collection and transport

Field conditions	The field conditions in the Fram Strait are typical of an Arctic marine environment, with a strong influence from climate variability. The region experiences marked seasonal and interannual fluctuations in temperature and sea ice cover, which in turn affect plankton community composition and the transport of organic matter.
Location	The sampling area was located in the Fram Strait. Pelagic samples were collected using Kiel-type sediment traps deployed at depths of 179–280 m within the water column at the central HAUSGARTEN station (79°01'N, 4°20'E). Sediment cores were retrieved from 19 stations along longitudinal (HG-I to HG-IX) and latitudinal (S3 to N5) transects. Additional stations regularly covered by sea ice (EG-I to EG-IV) in the western Fram Strait were also included in the study. The sampled region spanned a geographic range of 3°E–8°E and 78°5'N–80°N, with water depths ranging from 1000 m to 5500 m.
Access & import/export	The study area was accessed via research vessel, and samples were collected and exported in compliance with international laws. We apply for general research permits to the Directorate of Fisheries in Norway and the Ministry of Foreign Affairs in Denmark. Neither Denmark nor Norway requires Nagoya applications; instead, we simply notify them about our molecular studies. For the Exclusive Economic Zone (EEZ) of Greenland, however, we submit an annual application to the national contact point of Greenland for permission to conduct molecular analyses in compliance with the Nagoya Protocol. This specifically applies to stations within Greenland's EEZ: EGC4, EGC3, and EGC2.<br>It is important to emphasize the long-term observational nature of our study and that it is already granted that AWI holds all necessary permissions for these activities.
Disturbance	The study was conducted in the Fram Strait, a key Arctic region that is particularly sensitive to climate change. Environmental conditions such as temperature, sea ice cover, and seasonal variability are critical to the area and were highly relevant to our research.

Reporting for specific materials, systems and methods

We require information from authors about some types of materials, experimental systems and methods used in many studies. Here, indicate whether each material, system or method listed is relevant to your study. If you are not sure if a list item applies to your research, read the appropriate section before selecting a response.

Materials & experimental systems

n/a	Involved in the study
☒	☐ Antibodies
☒	☐ Eukaryotic cell lines
☒	☐ Palaeontology and archaeology
☒	☐ Animals and other organisms
☒	☐ Clinical data
☒	☐ Dual use research of concern
☒	☐ Plants

Methods

n/a	Involved in the study
☒	☐ ChIP-seq
☒	☐ Flow cytometry
☒	☐ MRI-based neuroimaging

Plants

Seed stocks	n/a
Novel plant genotypes	n/a
Authentication	n/a
